# A new variant of gallbladder duplication mimicking a choledochal cyst: stepwise management of an unexpected surgical finding

**DOI:** 10.1308/rcsann.2022.0131

**Published:** 2024-02-16

**Authors:** M Kim, S Lam, MA Thirunavaya Kalathil, A Paterson, DJ Bowden, SS Liau

**Affiliations:** Addenbrooke's Hospital, UK

**Keywords:** Choledochal cyst, Gallbladder duplication, Congenital, Cholecystectomy

## Abstract

We present a case of previously unclassified duplicated gallbladder which posed a surgical challenge intraoperatively by mimicking a choledochal cyst. An intraoperative cholangiogram was performed followed by a simple cholecystectomy. No further dissection was performed to avoid bile duct injury and complication from the unconventional anatomy. Postoperative imaging and histology, followed by the second operation confirmed findings consistent with the duplicated gallbladder. Through this case, we have demonstrated the principles of safe cholecystectomy and the importance of a staged approach in an unanticipated encounter of anatomical uncertainty, as well as the description of a new variant of duplicated gallbladder.

## Background

Duplication of gallbladder is a rare congenital malformation found in about 1:4,000 births and more than 200 cases have been reported according to a recent systematic review.^1^ This report highlights the case of a new variant of duplicated gallbladder that has not been previously classified by Harlafti’s classification. This case posed a clinical challenge intraoperatively by mimicking a choledochal cyst.

## Case history

**Figure 1:**
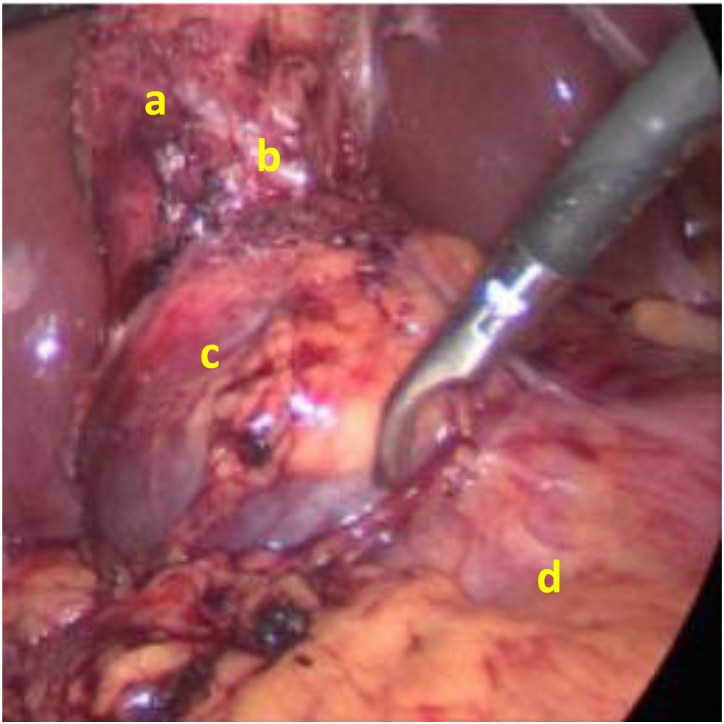
Intraoperative image showing (a) presumed first gallbladder, (b) cystic artery, (c) cystic structure and (d) duodenum.

A 44-year-old woman was referred for biliary colic with her recent ultrasound scan showing gallstones within a gallbladder and a non-dilated biliary tree with no history of jaundice. Laparoscopic cholecystectomy with intraoperative cholangiogram was performed. During the laparoscopy, a large cystic structure occupying the liver hilum was encountered (Figure 1). It appeared to occupy the position of the common bile duct (CBD) so the initial impression was a choledochal cyst.

Cholecystectomy was performed while leaving the presumed choledochal cyst undisturbed. Hartmann’s pouch was dissected to form the critical view of safety and wide space was created under the gallbladder belly. The gallbladder was opened to visualise cystic duct opening from the inside, which had the spiral valves of Heister. This was cannulated for cholangiogram which showed contrast filling of the cystic structure, but no drainage into the duodenum. At this stage, a simple cholecystectomy was performed to avoid further dissection. The presumed cystic duct was then clipped, and the gallbladder was resected. Given the uncertainty of the associated biliary anatomy, the situation was managed in a stepwise fashion – a simple cholecystectomy followed by further postoperative imaging to confirm the nature of the lesion and the associated anatomy, before proceeding to definitive management. The histology of the resected gallbladder confirmed chronic cholecystitis.

**Figure 2:**
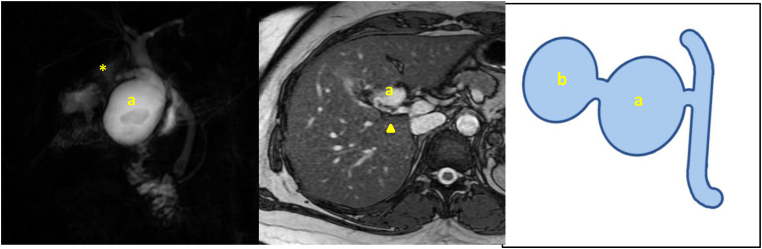
Postoperative magnetic resonance cholangiopancreatography images (left and middle) and illustration (right) showing (a) duplicated gallbladder and (b) first gallbladder (resected in the initial operation). The arrowhead indicates the cystic duct and the asterisk marks the cystic duct stump of the resected first gallbladder. Illustration drawn by M Kim.

Magnetic resonance cholangiopancreatography (MRCP) was undertaken postoperatively and showed a stone-containing cystic mass within the gallbladder fossa, communicating with the CBD and consistent with a duplicated gallbladder with a stone (Figure 2). In addition, the image reconstruction suggested the presence of a cystic stump of the first excised gallbladder. A stone in the CBD was also found and removed by endoscopic retrograde cholangiopancreatography.

**Figure 3:**
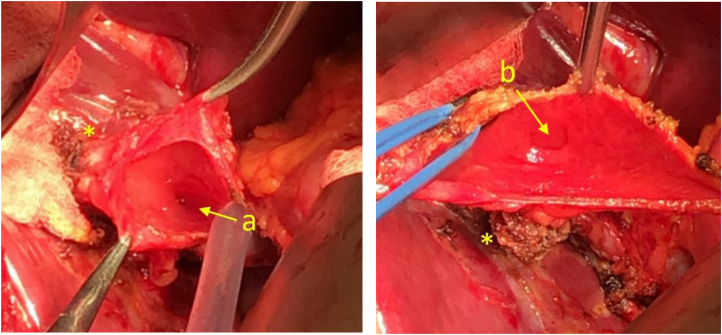
Gallbladder (opened) in the second operation showing the cystic duct opening draining from (a) the common bile duct and (b) the first gallbladder. The asterisk marks the cystic duct stump of the first gallbladder.

The patient remained asymptomatic but because she had remnant stones within the second gallbladder, the risks of future complications from gallstones were explained and a collaborative decision was made to undertake a second surgery to excise the second gallbladder. It was felt safest to plan an open surgery given the intimate relationships between the second gallbladder and the bile duct, to avoid inadvertent bile duct injury. A laparotomy for excision was performed 6 months later, when the second gallbladder was opened and two internal orifices – draining from the previously excised gallbladder and another to the CBD – were seen (Figure 3). Full intraoperative cholangiogram was achieved with visualisation of all the sectoral intrahepatic ducts. Histology further confirmed findings consistent with duplicated gallbladder. The patient made an uneventful recovery and was discharged on day 5 after operation.

## Discussion

Our case illustrates a duplicated gallbladder masquerading a choledochal cyst intraoperatively. Preoperative identification by ultrasound is understandably difficult because of the low index of suspicion and operator dependence of the technique.^2^ Ultrasound may pick up anomalies of the biliary system, but does not provide sufficient details of the cystic duct and CBD. The most useful imaging would be MRCP, which is not routinely performed if there is no suspicion of pathologies in the bile duct. It has been reported that only 50% of gallbladder duplication is diagnosed preoperatively.^3^ Presenting as an intraoperative surprise of unconventional anatomy, the challenge was to identify critical structures and complete the procedure safely. A fundus first gallbladder dissection to avoid an unclear Calot’s triangle, and intraoperative cholangiogram were the critical safety measures we adopted to avoid bile duct injury. Because the cystic structure was opacified in the cholangiogram, our initial impression was a choledochal cyst and further exploration was not pursued. With this approach, our patient was able to recover from the surgery without major complications, while further investigations were being arranged to resolve the anatomical uncertainties.

**Figure 4:**
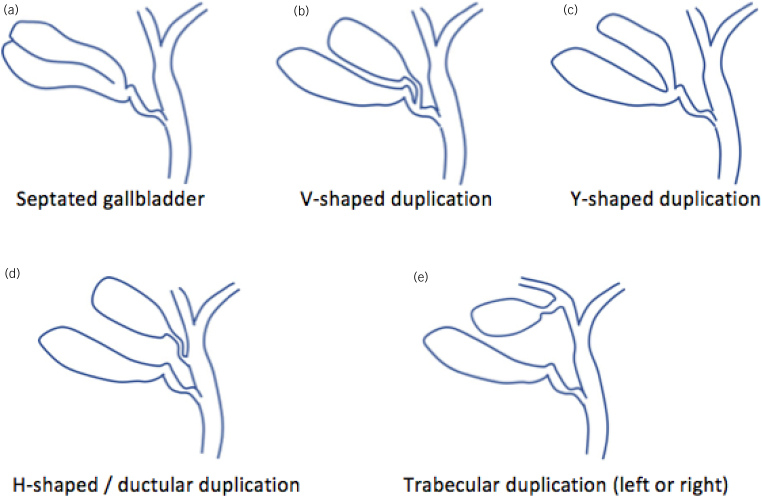
Classification of gallbladder duplication showing (a,b) Boyden’s vesica divisa type and (c,d) Boyden’s vesica fellea duplex type. Harlafti’s split primordium (type 1) is shown in (a)–(c) and Harlafti’s accessory gallbladder (type 2) is shown in (d) and (e). Illustration drawn by S Lam.

Gallbladder duplications were classified morphologically by Boyden in 1926 as bi-lobed gallbladder with a single cystic duct (vesica divisa type), or double gallbladder with a separate cyst duct (vesica fellea duplex type).^4^ Harlafti later proposed a classification system through literature review, addressing the embryological origin of malformation.^1^ In our case, the gallbladder arrangement only remotely resembles the V-shaped type of duplication (Figure 4).

It could also provide support for a previously undescribed variant case outside the classification system reported by Winkle, which demonstrates a similar anomaly as our case.^5^ Such rarity of the case contributed to the challenge not only in identifying on imaging preoperatively, but also in recognising the possibility of gallbladder duplication intraoperatively. We have demonstrated a safe stepwise approach to managing such unexpected intraoperative findings with an otherwise uncomplicated preoperative picture of biliary colic.

## Conclusion

This rare case of a possibly new variant of duplicated gallbladder demonstrates the importance of the value of fundus first dissection and intraoperative cholangiogram as a safety approach in unanticipated intraoperative encounter of anatomical uncertainty.
